# Oxidative Damage and Mitochondrial Injuries Are Induced by Various Irrigation Pressures in Rabbit Models of Mild and Severe Hydronephrosis

**DOI:** 10.1371/journal.pone.0127143

**Published:** 2015-06-19

**Authors:** Zhixiu Cao, Weimin Yu, Wei Li, Fan Cheng, Ting Rao, Xiaobing Yao, Xiaobin Zhang, Stéphane Larré

**Affiliations:** 1 Department of urology, Renmin hospital of Wuhan University, Wuhan, Hubei, China; 2 Department of anesthesiology, Renmin hospital of Wuhan University, Wuhan, Hubei, China; 3 Department of urology, Wuhan NO.1 hospital, Wuhan, Hubei, China; 4 Department of Urology, Robert Debré Teaching Hospital, University of Reims, Reims, France; University of Louisville, UNITED STATES

## Abstract

**Objective:**

We aimed to study whether tolerance to irrigation pressure could be modified by evaluating the oxidative damage of obstructed kidneys based on rabbit models experiencing different degrees of hydronephrosis.

**Methods:**

A total of 66 rabbits were randomly divided into two experimental groups and a control group. In the experimental groups, the rabbits underwent a surgical procedure inducing mild (group M, n=24) or severe (group S, n=24) hydronephrosis. In each experimental group, the rabbits were then randomly divided into 4 subgroups (M0-M3 and S0-S3) consisting of 6 rabbits each. Group 0 received no perfusion. Groups 1 through 3 were perfused with 20, 60 and 100 mmHg fluid, respectively. For the control group, after a sham operation was performed, the rabbits were divided into 4 subgroups and were perfused with fluid at 0, 20, 60 or 100 mmHg of pressure. Kidney injuries was evaluated by neutrophil gelatinase associated lipocalin (NGAL). Oxidative damage was assessed by analyzing superoxide dismutase (Mn-SOD) activity, malondialdehyde (MDA) levels, glutathione reductase (GR), catalase (CAT) and peroxide (H_2_O_2_) levels, mitochondrial injuries was assessed by mitochondrial membrane potential (MMP), the mitochondrial ultrastructure and tubular cell apoptosis.

**Results:**

In the experimental groups, all results were similar for groups 0 and 1. In group 2, abnormalities were observed in the S group only, and the kidneys of rabbits in group 3 suffered oxidative damage and mitochondrial injuries with increased NGAL, decreased Mn-SOD, GR and CAT,increased MDA and H_2_O_2_, lower levels of MMP, mitochondrial vacuolization and an increased apoptotic index.

**Conclusion:**

In rabbits, severely obstructed kidneys were more susceptible to oxidative damage and mitochondrial injury than mildly obstructed kidneys when subjected to higher degrees of kidney perfusion pressure.

## Introduction

Flexible ureterorenoscopy and percutaneous nephrolithotomy are widely used for the treatment of kidney stones and present several advantages over open procedures including reduced post-operational pain, minimal scar tissue and shorter hospitalization time [1,2]. These endourological procedures require effective fluid perfusion pressure to obtain a clear operational visual field and to flush out kidney stone fragments. Guohua [3] reported that when intrapelvic pressure exceeds 30 mmHg, pyelovenous lymphatic backflow could occur. Venous outflow obstruction decreases glomerular filtration rate [4] and, in some instances, can result in severe renal injury or acute kidney injury [5,6]. However, current recommendations suggest the maintenance of perfusion pressure between 50 mmHg and 300 mmHg [7,8], which includes a lower limit that exceeds the 30 mmHg that can cause kidney injury. We speculated that the high pressure itself might induce renal oxidative damage and secondary loss of renal function due to insufficient venous flow and compression of microvessels. Moreover, most patients diagnosed with kidney stones also present with some degree of hydronephrosis, which may lead to reduced renal function over time [9,10]. Hydronephrotic kidneys may be more susceptible to damage from an increase in kidney pressure during endourological procedures. In this study, we investigated whether tolerance to irrigation pressure differs based on the degree of hydronephrosis by evaluating oxidative damage indicators in rabbit kidneys that were obstructed to different extents.

## Materials and Methods

### Animals and Groups

A total of 66 New Zealand white rabbits (weight 2.2±0.2 kg) were purchased from Wuhan Institute of Biological Products Co., Ltd. (Wuhan, China). Our study protocol was approved by the institutional review board of Wuhan University. Animal care and treatment were conducted in accordance with the National Institutes of Health Guidelines for ethical animal research.

Rabbits were randomly assigned to one of two experimental groups or one control group. For the experimental groups, a surgical procedure inducing mild (group M, n = 24) or severe (group S, n = 24) hydronephrosis was performed. After the procedure, the rabbits were randomly assigned to one of 4 subgroups (M0 to M3 and S0 to S3) with each subgroup consisting of 6 rabbits. Group 0 received no perfusion, whereas Groups 1 through 3 were perfused with fluid at pressures of 20, 60 and 100 mmHg, respectively. For the control group (n = 18), rabbits were randomly assigned to one of 3 subgroups (Ctrl 1, Ctrl 2 and Ctrl 3) with each group consisting of 6 rabbits. After a sham operation was performed, the control rabbits were perfused with fluid at a pressure of 20, 60, or 100 mmHg. Kidney injuries was assessed by the expression of neutrophil gelatinase associated lipocalin (NGAL). Kidney oxidative damage was assessed by analyzing superoxide dismutase (Mn-SOD) activity, malondialdehyde (MDA) levels, glutathione reductase (GR) levels, catalase (CAT) and peroxide (H_2_O_2_) levels. Kidney mitochondrial injuries was assessed by mitochondrial membrane potential (MMP), mitochondrial ultrastructural changes, and tubular cell apoptosis.

### Surgical manipulation

Hydronephrosis was induced as previously described by Wen et al. [11]. Briefly, the rabbits were anesthetized via the marginal vein of the ear (30 mg/kg pentobarbital, iv). Animals were fixed in a supine position on an electric warming blanket to maintain normal body temperature. The left ureter, left lumbar vein and psoas muscle were exposed by a midline abdominal incision. For the mild hydronephrosis group, the upper fourth of the left ureter was buried in the psoas muscle, and the edges of the muscle were then sutured over the ureter. For the severe hydronephrosis group, a similar procedure was performed using the upper two-thirds of the left ureter. The rabbits in the control group underwent the midline abdominal incision to expose the left ureter, left lumbar vein and psoas muscle without burying the ureters. Two weeks after the surgery, B-ultrasonography was used to confirm hydronephrosis. In The M and S groups respectively, pyelic distention was 0.95±0.27 cm and 1.69±0.34cm and thickness of parenchyma was 0.33±0.09 cm and 0.22±0.05 cm. These parameters were statistically different between (p<0.05). After hydronephrosis was confirmed, a second laparotomy was performed. The hydronephrotic kidney was surgically exposed, and a 0.7-gauge intravenous pediatric scalp needle was inserted into the renal collecting system through the parenchyma to a depth of 4–5 mm. A physiological recorder (Biopac, MP150, US) was connected to the needle to record the intrapelvic pressure. Another 4-5-mm-deep tract was dilated, and then a 1 mm diameter steel needle was inserted into the tract, to which a pressure pump was connected (Laborie, UDS64-III, Switzerland). A 37°C isotonic saline solution was used for perfusion. The M0 group and the S0 groups were punctured but were not perfused. Before perfusion, the intrapelvic pressures were measured in mild and severe hydronephrosis kidneys, they were respectively 10.48±1.95 mmHg and 15.86±2.87 mmHg. These differences were statistically significant (p<0.05). Then groups 1, 2 and 3 were perfused at 20, 60 and 100 mmHg, respectively, for 8 minutes. After 8 minutes, the perfusion was stopped for 2 minutes. This cycle was repeated 4 times. After the perfusion, the psoas muscle obstruction was relieved, and the abdomen was sutured closed. Rabbits were sacrificed using 150 mg/kg pentobarbital (20%) through the ear marginal vein injection after 48 h, and the left kidneys were collected for biochemical and histological evaluation.

### Immunohistochemical staining and evaluation

Tissues were fixed in 10% buffered formalin overnight. Then, the tissues were embedded in paraffin blocks from which 5-μm-thick sections were cut. The tissue slides were deparaffinized by submerging the slides in xylene and then were rehydrated by using a graded ethanol series. After microwave antigen retrieval and inactivating endogenous enzymes, slides were incubated with primary antibodies NGAL (Santa Cruz Biotechnology, CA) over night in a humidified chamber at 4°C. Then biotinylated secondary antibodies, horseradish peroxidase and 3,30-diaminobenzidine (Zhongshan Golden Bridge Biotechonlogy, Beijing, China) were added as the manufacture’s introduction. After being counterstained with hematoxylin, the sections were purified with hydrochloric acid alcohol, dehydrated in a graded ethanol series, cleaned in xylene, and mounted in gelatin. Immunostaining scores were evaluated by adding the density and intensity scores [12]. The density score was obtained as the percentage of positive staining cells on each section. Sections were graded as follows: 0 (no staining), 1 (staining<25%), 2 (staining between 25% and 50%), 3 (staining between 50% and 75%), and 4 (staining>75%). The intensity score was graded as follows: 0 (no staining), 1 (weak but detectable staining), 2 (distinct staining), and 3 (intense staining). All slides were imaged and quantified by 3 pathologists in a blinded manner through Olympus IX 70 microscope (Olympus Optical, Tokyo, Japan).

### Superoxide dismutase, glutathione reductase, catalase malondialdehyde and peroxide measurement

Tissues were homogenized by an Ultra-Turrax (T25, IKAH-Labortechnik, Staufen, Germany) in 20 mmol/L Tris buffer (pH 7.4) containing 5 mM butylated hydroxytoluene (BHT) to prevent new lipid peroxidation that can occur during homogenization. The homogenate was centrifuged at 12000 × g at 4°C (Heraeus Biofuge Primo R, Karlsruhe, Germany), and the supernatant was used to measure Mn-SOD and MDA activity levels. Mn-SOD, GR, CAT, MDA and H_2_O_2_ were examined using standard assay kits (Nanjing Jiancheng Bioengineering Institute, Jiangsu, China) according to the manufacturer’s instructions. One unit of Mn-SOD was defined as enough protein for 50% inhibition of the rate of reduction based on the conditions of the assay. The activity of Mn-SOD was expressed as U/mg. The level of GR, CAT,MDA, and H_2_O_2_ were calculated based on the absorbance coefficient of the supernatant complex and was expressed as mg/L, U/ml, μmol/g and nM/mg·ml^-1^.

### Mitochondrial membrane potential detection

Mitochondrial membrane potential (MMP) was determined by using the 5,5’,6,6’-tetrachloro-1,1’,3,3’-tetraethylbenzimidazolycarbocyanine iodide (JC-1, Beyotime Institute of Biotechnology, Jiangsu, China). Briefly, renal tissue was digested in a trypsin-EDTA solution (Beyotime Institute of Biotechnology, Jiangsu, China), and then the digestion was terminated by adding bovine serum. Suspension cells were harvested and were loaded with 1×JC-1 at 37°C for 20 min, then the cells were washed and analyzed by flow cytometry (FACS Aria III, BD, New Jersey, US). JC-1 is a membrane-permeable lipophilic dye that exists as J-aggregates in the mitochondrial matrix (red fluorescence) and as monomers in the cytoplasm (green fluorescence). During mitochondrial depolarization, the red J-aggregates form green monomers due to a change in ΔΨ. Thus, mitochondrial membrane potential can be measured as an increasing green fluorescent/red fluorescent intensity ratio. When MMP levels are low, JC-1 exists mainly as a monomer, which emits green fluorescence (excitation wavelength of 490 nm and emission wavelength of 540 nm). When MMP levels are high, JC-1 exists mainly as a polymer, which emits red fluorescence (excitation wavelength of 525 nm and emission wavelength of 590 nm). [13–15] The value of MMP was expressed as the ratio of red fluorescence intensity to green fluorescence intensity.

### Kidney ultrastructure by electron microscopy

Dissected tissues were fixed with 2.5% glutaraldehyde, rinsed three times in 0.1 M phosphate buffer, postfixed in 1% buffered osmium tetroxide and then rinsed in 0.1 M phosphate buffer. Subsequently, tissues were dehydrated by incubating the tissue in graded alcohol, and the dehydrated tissue was embedded in epoxy resin. Thin tissue sections were cut by using an ultramicrotome (LKB, Bromma, Kista, Sweden) and were stained with uranyl acetate and lead citrate. Ultrastructural changes were observed and were photographed using a transmission electron microscope (H-600, Hitachi, Tokyo, Japan).

### Apoptosis detection

Tissues were fixed in 10% buffered formalin overnight. Then, the tissues were embedded in paraffin blocks from which 5-μm-thick sections were cut. The tissue slides were deparaffinized by submerging the slides in xylene and then were rehydrated by using a graded ethanol series. Apoptosis was assessed by using the terminal deoxynucleotidyl transferase-mediated deoxyuridine triphosphate-biotin nick-end labeling (TUNEL) with the In Situ Apoptosis Detection kit (Roche Applied Science, Basel, Switzerland). The apoptotic index was calculated based on the percentage of positive apoptotic nuclei in five high-power fields of view per tissue section).

### Statistical Analysis

All data are expressed as the means ± SD. Statistical significance was assessed by ANOVA with subsequent S-N-K test using SPSS (19.0) software. For all analysis, p-values less than 0.05 were considered statistically significant.

## Results

### NGAL Expression of hydronephrotic kidneys after perfusion

**Fig 1:**
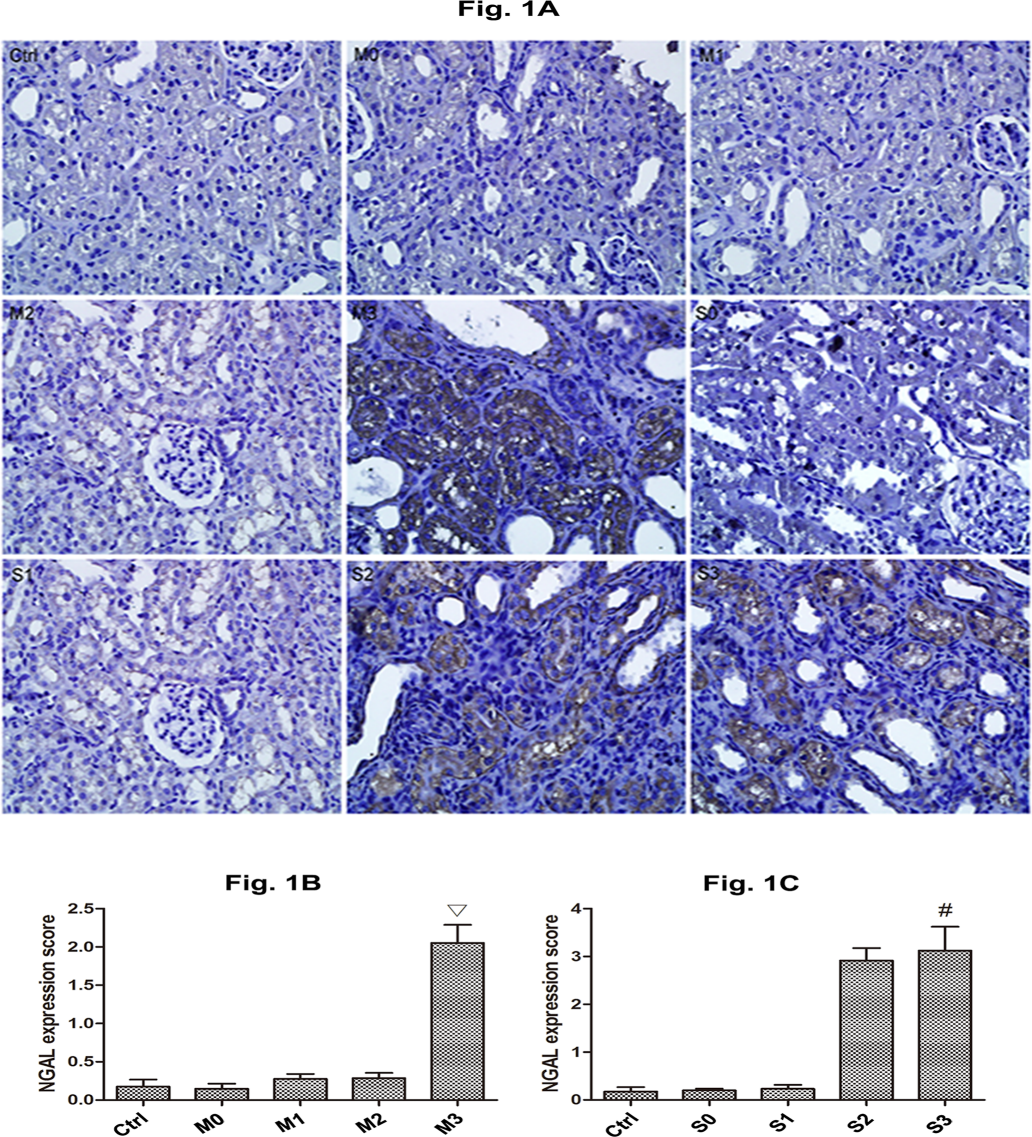
Expression of NGAL in the kidneys perfused at different pressures in rabbits with mild and severe hydronephrosis. A: NGAL expression (×400), the brown fields in the cytoplasm represented NGAL expression. B: Immunostaining scores of NGAL with different perfusion pressure in mildly kidneys (M group). C: Immunostaining scores of NGAL with different perfusion pressure in severely obstructed kidneys (S group). The bars represent means±SE; ▽p<0.05 compared with the M0 group. #p<0.05 compared with the S0 group. Ctrl: normal kidneys perfused with 20 mmHg, 60 mmHg or 100 mmHg. M0, M1, M2, and M3 represent rabbits with mild hydronephrosis subjected to perfusion pressure of 0 mmHg, 20 mmHg, 60 mmHg and 100 mmHg, respectively. S0, S1, S2, S3 represent rabbits with severe hydronephrosis subjected to perfusion pressure of 0 mmHg, 20 mmHg, 60 mmHg and 100 mmHg, respectively.

Significant cytoplasmic expression of NGAL was induced by high perfusion (Fig 1A). In M group, NGAL expression was similar at 0, 20 and 60 mmHg (Fig 1B, p>0.05), but increased at 100 mmHg (Fig 1B, p< 0.05). In S group, NGAL expression was similar at 0 and 20 mmHg (Fig 1C), but increased at 60 and 100 mmHg (Fig 1C). No significant difference in NGAL expression were observed between S2 and S3 groups (p>0.05).

### Effect of perfusion of hydronephrotic kidneys on Mn-SOD GR, CAT, MDA and H2O2 content in tissues

**Fig 2:**
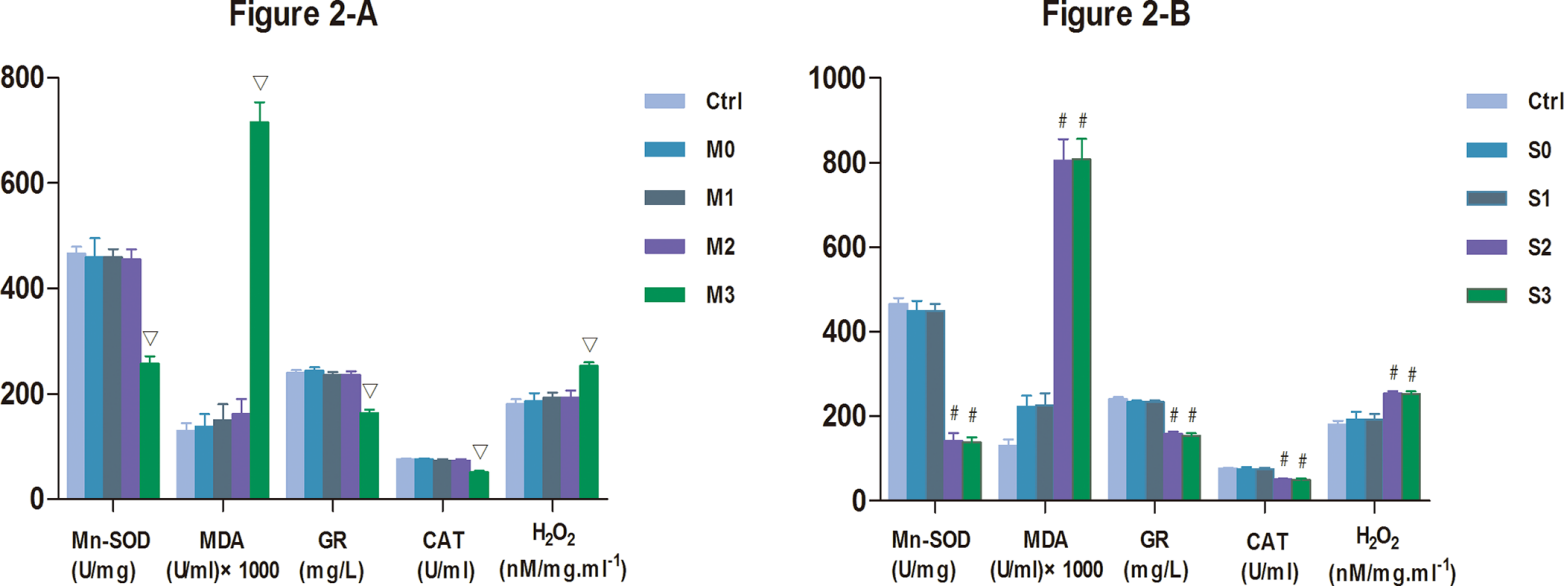
Levels of Mn-SOD, GR, and CAT, MDA and H_2_O_2_ in the kidneys perfused at different pressures in rabbits with mild and severe hydronephrosis. A: Activity of Mn-SOD, GR, CAT, MDA, H_2_O_2_ in rabbits with mild hydronephrosis. B: Activity of Mn-SOD, GR, CAT, MDA, H_2_O_2_ in rabbits with severe hydronephrosis.▽p<0.05 compared with the M0 group. #p<0.05 compared with the S0 group. Ctrl: normal kidneys perfused with 20 mmHg, 60 mmHg or 100 mmHg. M0, M1, M2, and M3 represent rabbits with mild hydronephrosis subjected to perfusion pressure of 0 mmHg, 20 mmHg, 60 mmHg and 100 mmHg, respectively. S0, S1, S2, S3 represent rabbits with severe hydronephrosis subjected to perfusion pressure of 0 mmHg, 20 mmHg, 60 mmHg and 100 mmHg, respectively.

High perfusion resulted in decreased levels of Mn-SOD, GR, CAT and increase levels of MDA and H_2_O_2_ in renal tissues of group M3, S2 and S3(Fig 2A and Fig 2B). Mn-SOD, GR, CAT, MDA and H_2_O_2_ levels were comparable in Ctrl 1, Ctrl 2 and Ctrl 3, and thus, we combined these groups for our analysis. Mn-SOD, GR, CAT, MDA and H_2_O_2_ levels levels in the rabbits with mild hydronephrosis perfused with fluid at pressures of 0 mmHg, 20 mmHg and 60 mmHg were also comparable, They were also comparable in rabbits with severe hydronephrosis perfused with fluid at pressures of 0 mmHg and 20 mmHg (p>0.05, Fig 2B). In rabbits with mild hydronephrosis, Mn-SOD, GR and CAT levels decreased when the perfusion pressure increased to 100 mmHg (p<0.05, Fig 2A), however, the MDA and H_2_O_2_ levels increased in this group when subjected to a perfusion pressure of 100 mmHg (p<0.05, Fig 2A). In rabbits with severe hydronephrosis, the Mn-SOD, GR and CAT levels decreased when subjected to perfusion pressure of 60 mmHg or 100 mmHg (p<0.05, Fig 2B); however, the MDA and H_2_O_2_ levels in this group increased when subjected to perfusion pressure of 60 mmHg or 100 mmHg (p<0.05, Fig 2B).

### Effect of perfusion of hydronephrotic kidneys on MMP levels

**Fig 3:**
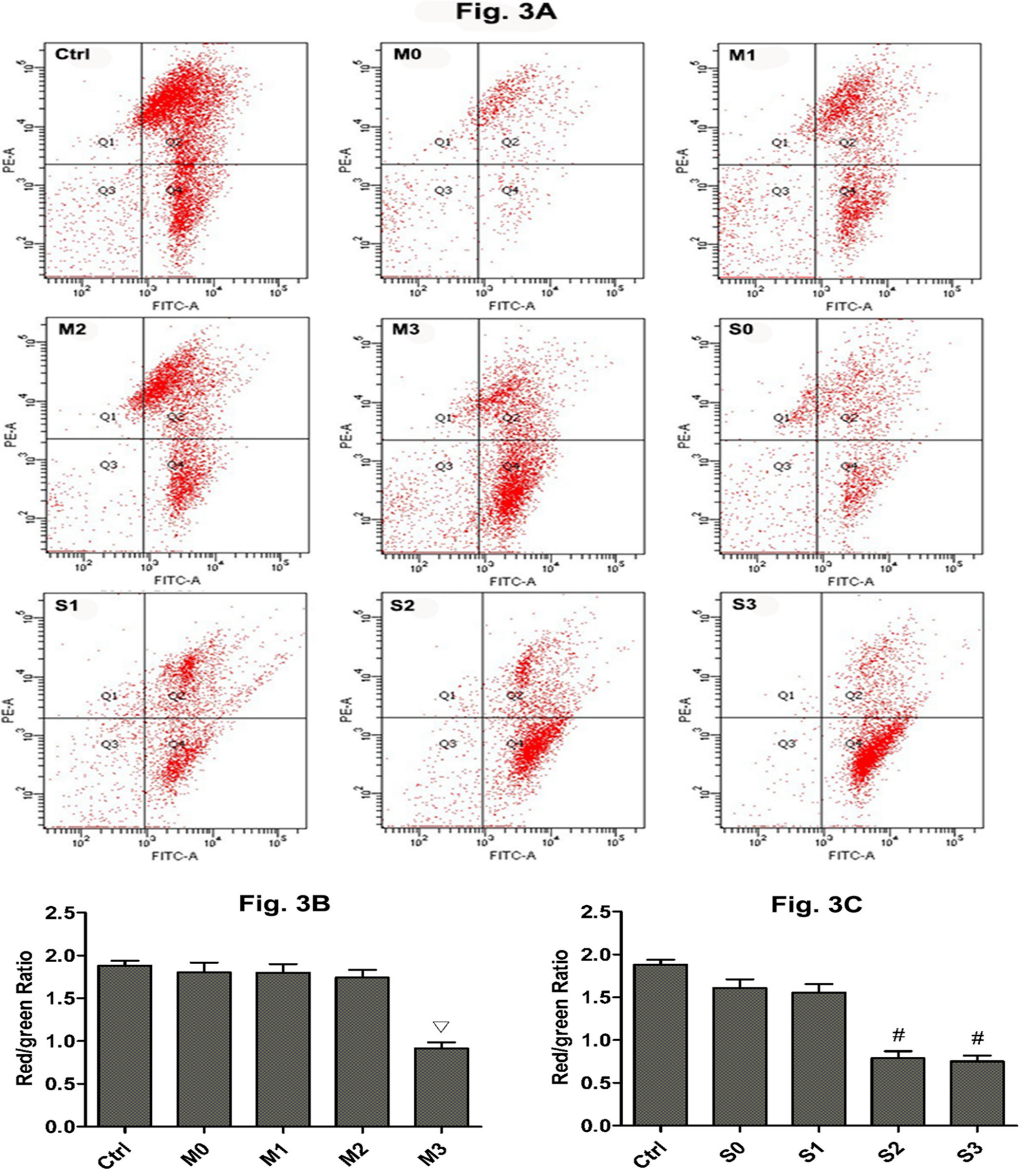
Mitochondrial membrane potential (MMP) of renal cells subjected to different amounts of pressure in mild and severe hydronephrosis. PE-A Represented red fluorescence, FITC-A represented green fluorescence. MMP values were expressed as the ratio of red fluorescence intensity (Q2) to the green fluorescence intensity (Q4). A: MMP analysis by flow cytometry in rabbits with mild and severe hydronephrosis. B: MMP of renal cells in rabbits with mild hydronephrosis. C: MMP of renal cells in rabbits with severe hydronephrosis. ▽p<0.05 compared with the M0 group. #p<0.05 compared with the S0 group. Ctrl: normal kidneys perfused with 20 mmHg, 60 mmHg and 100 mmHg. M0, M1, M2, and M3 represent rabbits with mild hydronephrosis subjected to perfusion pressure of 0 mmHg, 20 mmHg, 60 mmHg and 100 mmHg, respectively. S0, S1, S2, and S3 represent rabbits with severe hydronephrosis subjected to perfusion pressure of 0 mmHg, 20 mmHg, 60 mmHg and 100 mmHg, respectively.

A decrease in MMP levels is considered an event of early apoptosis. To better illustrate the dysfunction of mitochondria in kidneys subjected to high perfusion pressure, MMP was measured in kidneys by using flow cytometry and JC-1 staining. A decrease in MMP is indicated by a decreased in the ratio of red fluorescence (JC-1 polymer) to green fluorescence (JC-1 monomer). Fig 3A shows the ratio of red fluorescence and green fluorescence of renal cells as determined by flow cytometry. Red fluorescence decreased and green fluorescence increased as the perfusion pressure in obstructed kidneys increased. MMP was higher in the control group than in all mild and severe hydronephrosis groups (p<0.05). Furthermore, all control groups were similar with respect to MMP; thus, we grouped them together. In rabbits with mild hydronephrosis, the MMP levels were similar at 0 mmHg, 20 mmHg, and 60 mmHg (p>0.05) but were significantly lower at 100 mmHg (p<0.05, Fig 3B). In rabbits with severe hydronephrosis, the MMP levels were similar at 0 mmHg and 20 mmHg but were significantly lower at 60 mmHg and 100 mmHg (p<0.05, Fig 3C). In rabbits with severe hydronephrosis, the MMP levels were similar between the 60 mmHg and 100 mmHg subgroups (p>0.05, Fig 3C).

### Changes in the ultrastructure of the mitochondria

**Fig 4:**
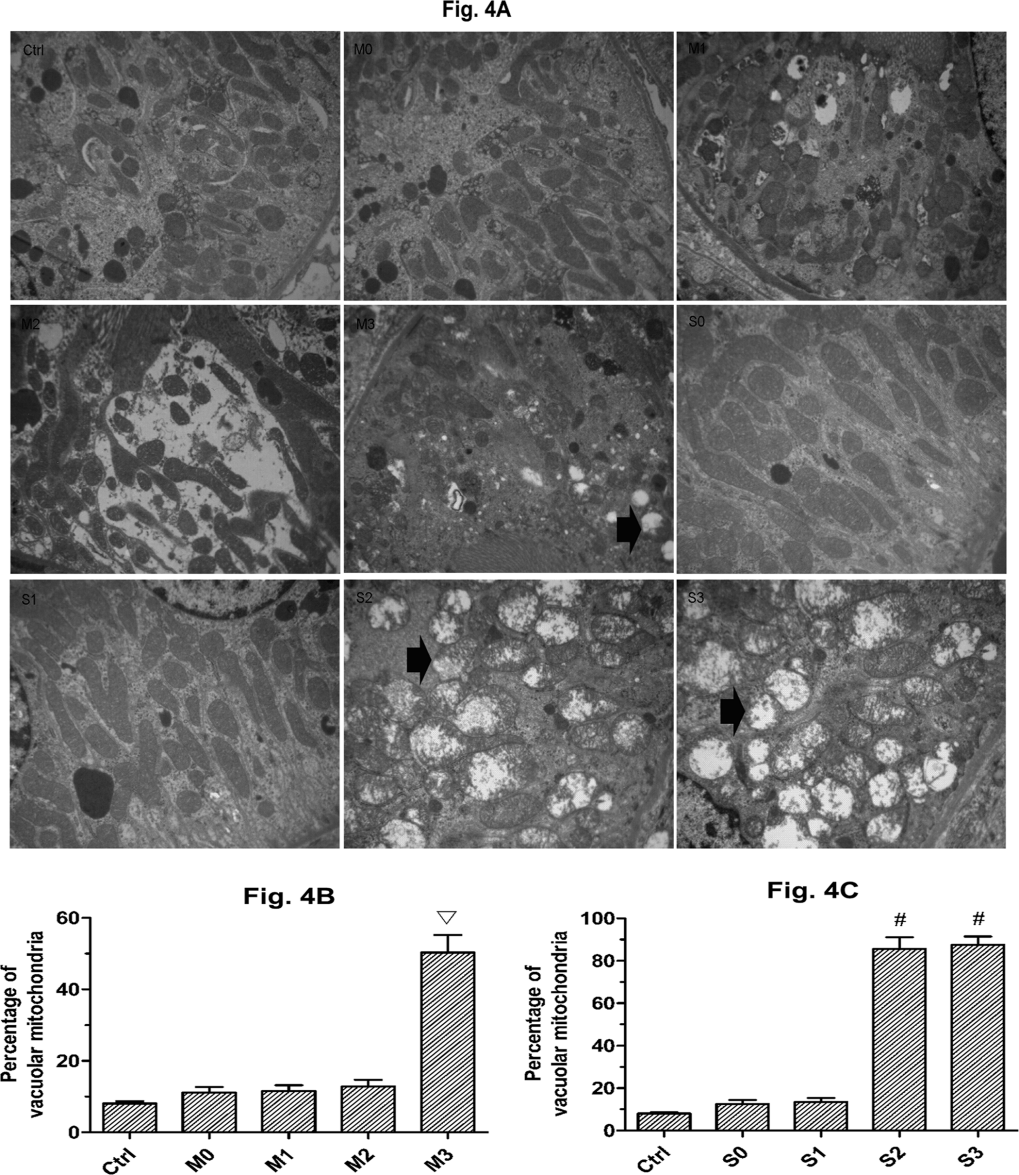
Ultrastructural changes of the mitochondria. A: Swollen and vacuolar mitochondria in rabbits with mild and severe hydronephrosis under different perfusion pressures (×10000), the arrows showed the swollen and vacuolar mitochondria. B: Percentage of swollen and vacuolar mitochondria in rabbits with mild hydronephrosis. C: Percentage of swollen and vacuolar mitochondria in rabbits with severe hydronephrosis. ▽p<0.05 compared with the M0 group. #p<0.05 compared with the S0 group. Ctrl: normal kidneys perfused with fluid at 20 mmHg, 60 mmHg and 100 mmHg. M0, M1, M2, and M3 represent rabbits with mild hydronephrosis subjected to perfusion pressure of 0 mmHg, 20 mmHg, 60 mmHg and 100 mmHg, respectively. S0, S1, S2, and S3 represent rabbits with severe hydronephrosis subjected to perfusion pressure of 0 mmHg, 20 mmHg, 60 mmHg and 100 mmHg, respectively.

To investigate the mitochondrial damage in the tubular cells in kidneys stimulated by perfusion pressure, the percentage of swollen and vacuolar mitochondria were counted in five random fields of view for each section. Fig 4A shows the swollen and vacuolar mitochondria at different perfusion pressures in rabbits with mild and severe hydronephrosis. In the control groups, the amount of swollen and vacuolar mitochondria was much lower than in all other groups, Furthermore, all of the control groups were comparable, and thus we grouped them together for our analysis. In rabbits with mild hydronephrosis, the percentage of swollen and vacuolar mitochondria was similar at 0 mmHg, 20 mmHg, and 60 mmHg (p>0.05) but was significantly increased at 100 mmHg (p<0.05, Fig 4B). In rabbits with severe hydronephrosis, the percentage of swollen and vacuolar mitochondria was similar at 0 mmHg and 20 mmHg but was significantly increased at 60 mmHg and 100 mmHg (p<0.05, Fig 4C).

### Apoptotic index analysis

**Fig 5:**
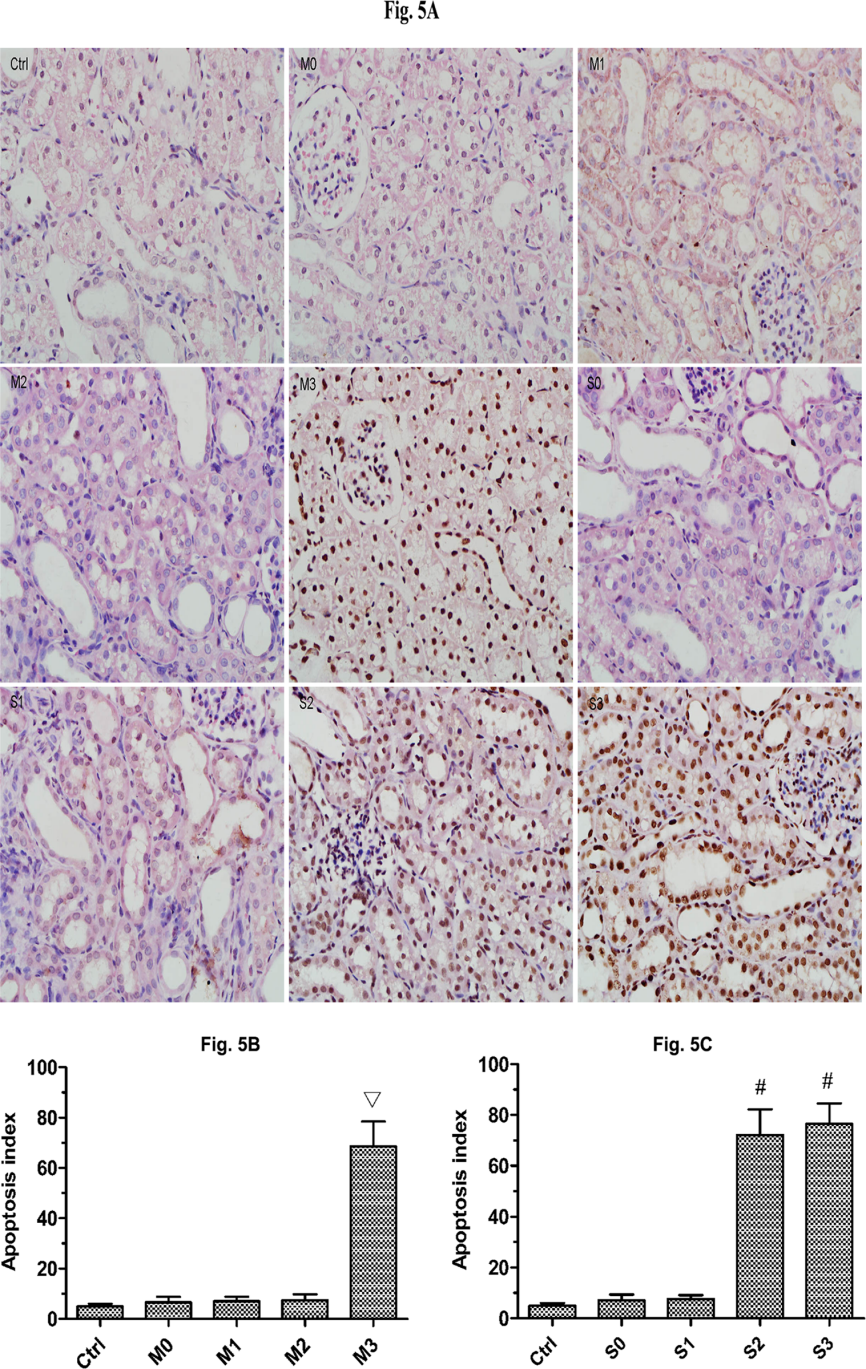
Apoptosis in rabbits with mild and severe hydronephrosis subjected to different perfusion pressures. A: Apoptosis of renal cells in rabbits with mild and severe hydronephrosis after being perfused with fluid at different pressures (×400). B: Percentage of apoptotic cells in rabbits with mild hydronephrosis. C: Percentage of apoptotic cells in rabbits with severe hydronephrosis. ▽p<0.05 compared with the M0 group. #p<0.05 compared with the S0 group. Ctrl: normal kidneys perfused with 20 mmHg, 60 mmHg and 100 mmHg. M0, M1, M2, and M3 represent rabbits with mild hydronephrosis subjected to perfusion pressure of 0 mmHg, 20 mmHg, 60 mmHg and 100 mmHg, respectively. S0, S1, S2, and S3 represent rabbits with severe hydronephrosis subjected to perfusion pressure of 0 mmHg, 20 mmHg, 60 mmHg and 100 mmHg, respectively.

Significant tubular apoptosis was induced by high-pressure perfusion (Fig 5). Apoptotic cells were observed less frequently in the control groups (Fig 5A), and the apoptotic index of the control groups was lower than all mild and severe groups (p<0.05). We observed no significant differences among the control groups, and thus we grouped them together for our analysis. In rabbits with mild hydronephrosis, the apoptotic index was similar at 0 mmHg, 20 mmHg and 60 mmHg (p>0.05) but was significantly increased at 100 mmHg (p<0.05, Fig 5B). In rabbits with severe hydronephrosis, the apoptotic index was similar at 0 mmHg and 20 mmHg but was significantly increased at 60 mmHg and 100 mmHg (p<0.05, Fig 5C). The tubular apoptotic index in rabbits with severe hydronephrosis was similar between the 60 mmHg and 100 mmHg groups (p>0.05, Fig 5C).

## Discussion

High pressure is often used during endourological interventions, however, our results show that oxidative damage can occur above a specific pressure threshold. Our results also show that oxidative damage occurs more often in kidneys that show a greater extent of obstruction. These results indicate that kidneys with severe hydronephrosis might have a decreased capacity to withstand perfusion pressure and could more easily suffer oxidative damage during endourological procedures.

In our study, the surgical conditions of endourological human procedures were imitated in rabbits. Our models are especially representative of flexible ureterorenoscopy and percutaneous nephrolithotripsy; however, inherent limitations related to our animal models exist. A potential limitation is that the perfusion pressures used in our study may not be physiologically relevant to the kidney perfusion pressures that could be applied in humans due to differences in kidney cell tolerance. Furthermore, the level of obstruction was not quantified in our study, and the level of obstruction might be different from the obstruction observed in humans. Whether certain kidney injuries due to perfusion can be reversed or whether perfusion injury would result in a permanent decline in kidney function remains unknown. However, our results demonstrated that kidney oxidative damage and mitochondrial injuries were more likely to occur in obstructed kidneys and that these damage are more extensive as the obstruction becomes more severe. Our results also suggest that further studies should be conducted to confirm the optimal kidney perfusion pressure that should be used to prevent kidneys from experiencing oxidative damage while maintaining an adequate field of view and sufficient pressure to remove kidney stones. Though a maintainence pressure of 50–300 mm Hg was recommended human [7,8], rabbits kidneys oxidative damage and mitochondrial injuries could occurred when the perfusion pressure exceeded 100 mmHg and 60 mmHg respectively in mild and severe hydronephrosis kidneys of our study.

Renal venous outflow obstruction is more detrimental to kidneys than arterial obstruction due to ischemia/reperfusion injury [6,16]. Standard perfusion pressure (50–300 mmHg) is typically used for endourological procedures, which might cause high intrapelvic pressure [3, 17,18]. However, intrapelvic pressure that exceeds 30 mmHg may induce pyelovenous backflow [3]. Pyelovenous backflow limits venous outflow to a certain extent, and renal microvessels become compressed by the perfusion pressure, which decreases the blood supply to the renal parenchyma. Both of these factors lead to ischemia/reperfusion damage to the kidney.

NGAL is a sensitive biomarker during the process of acute kidney injury. It occurred in proximal tubule within 3 h after kidney injury, then it relase to plasma within 12 h [19]. In our study, increased NGAL expression were observed in rabbits with mild hydronephrosis subjected to 100 mmHg perfusion pressure and in rabbits with severe hydronephrosis subjected to 60 and 100 mmHg perfusion pressure. These results suggest that the renal tubule was damage when kidneys are severely obstructed relative to kidneys that are mildly obstructed.

Reactive oxygen species (ROS) are mediators of tissue damage such as ischemic injuries and inflammatory insults, and ROS accumulation is extremely detrimental to cellular health [20–21]. Superoxide dismutase (SOD) is an antioxidant enzyme capable of transforming free radicals or reactive oxygen species intermediates into non-radical products, which consequently protects tissues against the toxic effects of superoxide radicals [22–23], Mn-SOD is highly expressed in mitochondria. It is critical for mitochondria suppression of oxidative damage, it could remove toxic superoxide anions in mitochondrial matrix and prevent superoxide anions from damage electron transport chain [24–26]. The antioxidant potential in tissue may be determined by using the Mn-SOD, GR, and CAT levels as a surrogate. Malondialdehyde (MDA) is a product of oxidative stress-mediated lipid peroxidation in renal tissue,it causes significant mitochondrial dysfunction by inhibition of respiration and inactivation of important enzymes in the mitochondria, such as Pyruvate dehydrogenase (PDH), α-Ketoglutaric dehydrogenase (KGDH), complexes I and II. MDA could also increase mitochondrial decay, especially, oxidative mitochondrial damage, including lipid peroxidation, protein oxidation and DNA damage [27,28] and the damage that ROS impart on tissue could be quantitatively shown by using MDA levels as a surrogate [29]. During the process of oxidative stress, hydrogen peroxide (H_2_O_2_) is an important member of ROS, glutathione (GSH) reacts with H_2_O_2_ formed glutathione disulfide (GSSG), glutathione reductase (GR) sustains the GSH/GSSG cycle to facilitate the transfer of electrons from glucose to H_2_O_2_ to eliminate H_2_O_2,_ thus preventing oxidative damage [30]. Catalase is also is an important endogenous antioxidant enzyme that catalyzes H_2_O_2_ detoxification [31]. In our study, decreased Mn-SOD, GR, CAT levels and increased MDA, H_2_O_2_ levels were observed in rabbits with mild hydronephrosis subjected to 100 mmHg perfusion pressure and in rabbits with severe hydronephrosis subjected to 60 and 100 mmHg perfusion pressure. These results suggest that the ability to tolerate perfusion pressure decreases when kidneys are severely obstructed relative to kidneys that are mildly obstructed. However, the Mn-SOD, GR, CAT levels were higher and the MDA, H_2_O_2_ levels were lower in all hydronephrotic groups relative to the control rabbits, suggesting that normal kidneys show better tolerance for perfusion pressure.

Maintaining the mitochondrial membrane potential (MMP) is critical for cellular viability [32]. A loss of MMP causes the opening of the mitochondrial permeability transition pore (mPTP) and subsequent mitochondrial content leakage. Due to the opening of the mPTP, apoptosis-activating factors such as cytochrome c are released, and the intrinsic pathway of apoptosis is triggered [33–35]. A decrease in MMP is considered indicative of early apoptosis. In our study, the MMP decreased in the mild hydronephrosis group upon exposure to perfusion pressure of 100 mmHg, and the MMP decreased in the severe hydronephrosis group upon exposure to perfusion pressure of 60 and 100 mmHg. These results are consistent with the apoptosis indicators, the apoptotic index increased in the mild hydronephrosis group when subjected to perfusion pressure of 100 mmHg, and the apoptotic index increased in the severe hydronephrosis group when subjected to perfusion pressure of 60 and 100 mmHg.

We also observed an increase in mitochondria that were swollen and vacuolar in the mild hydronephrosis group subjected to perfusion pressure of 100 mmHg, and we observed an increase in damaged mitochondria in the severe hydronephrosis group subjected to perfusion pressure of 60 and 100 mmHg. As cells lose the MMP, the influx of small molecules leads to mitochondrial swelling and vacuolization, which induces mitochondrial collapse followed by the leakage of cytochrome c, cell death and an ATP production decrease [36,37]. When accompanied by mitochondrial damage, the tolerance of obstructed kidneys to perfusion pressure decreases due to an increase of apoptotic cells and a decrease in ATP production.

Our study suggests that normal kidneys are able to better tolerate perfusion pressure than obstructed kidneys, and severely obstructed kidneys are more poorly tolerant of perfusion pressure than mildly obstructed kidneys. Hydronephrosis results in tubular dilatation, glomerulosclerosis, a decrease in renal blood flow, inflammation and subsequent fibrosis [38–40]. We speculated that differences in tolerance to perfusion pressure in obstructed kidneys was connected with the extent of obstruction in the kidneys; thus, based on our results, the mechanisms responsible for the decrease in tolerance to perfusion pressure should be further studied.

## Conclusion

In rabbits, severely obstructed kidneys suffer more intense oxidative damage and mitochondrial injury than mildly obstructed kidneys in the presence of perfusion pressure. Pressure should be maintained at the lowest effective level possible during processes such as flexible ureterorenoscopy or percutaneous nephrolithotripsy.

## Supporting Information

S1 Excel Original data. All of the original experiment data of the article were contained in the S1 Excel.
(XLS)

## References

[1] Elashry OM, Elgamasy AK, Sabaa MA, Abo-Elenien M, Omar MA, Eltatawy HH, et al (2008) Ureteroscopic management of lower ureteric calculi:a 15-year single-centre experience.BJU Int 102(8):1010–7. 10.1111/j.1464-410X.2008.07747.x 18485033

[2] Wu W, Zhao Z, Zhu H, Yang D, Ou L, Liang Y, et al (2014) Safety and efficacy of minimally invasive percutaneous nephrolithotomy in treatment of calculi in horseshoe kidneys. J Endourol 28(8): 926–9. 10.1089/end.2013.0760 24716526

[3] Guohua Z, Wen Z, Xun L, Wenzhong C, Yongzhong H, Zhaohui H, et al (2007) The influence of minimally invasive percutaneous nephrolithotomy on renal pelvic pressure in vivo. Surg Laparosc Endosc Percutan Tech 17(4): 307–310. 17710055 10.1097/SLE.0b013e31806e61f4

[4] Joles JA, Bongartz LG, Gaillard CA, Braam B. (2009) Renal venous congestion and renal function in congestive heart failure. J Am Coll Cardiol 54(17):1632–3 10.1016/j.jacc.2009.05.068 19833266

[5] Park Y, Hirose R, Dang K, Xu F, Behrends M, Tan V, et al (2008) Increased severity of renal ischemia-reperfusion injury with venous clamping compared to arterial clamping in a rat model. Surgery 143(2):243–51. 10.1016/j.surg.2007.07.041 18242341

[6] Li X, Liu M, Bedja D, Thoburn C, Gabrielson K, Racusen L, et al (2012) Acute renal venous obstruction is more detrimental to the kidney than arterial occlusion: implication for murine models of acute kidneyinjury. Am J Physiol Renal Physiol. 302(5): F519–25. 10.1152/ajprenal.00011.2011 22114209 PMC3353642

[7] Shao Y, Shen ZJ, Zhu YY, Sun XW, Lu J, Xia SJ et al (2012) Fluid-electrolyte and renal pelvic pressure changes during ureteroscopic lithotripsy. Minim Invasive Ther Allied Technol 21(4): 302–6. 10.3109/13645706.2011.595419 21745133

[8] Landman J, Venkatesh R, Ragab M, Rehman J, Lee DI, Morrissey KG, et al (2002) Comparison of intrarenal pressure and irrigant flow during percutaneous nephroscopy with an indwelling ureteral catheter, ureteral occlusion balloon, and ureteral access sheath. Urology 60(4):584–7. 12385911 10.1016/s0090-4295(02)01861-7

[9] Worcester EM, Parks JH, Evan AP, Coe FL. (2006) Renal function in patients with nephrolithiasis. J Urol 176(2):600–3 16813897 10.1016/j.juro.2006.03.095

[10] Kukreja R, Desai M, Patel SH, Desai MR. (2003) Nephrolithiasis associated with renal insufficiency: factors predicting outcome. J Endourol 17(10):875–9. 14744353 10.1089/089277903772036181

[11] Wen JG, Chen Y, F Frøkiaer J, Jørgensen TM, Djurhuus JC. (1998) Experimental partial unilateral ureter obstruction. I. Pressure flow relationship in a rat model with mild and severe acute ureter obstruction.J Urol 160(4):1567–71. 9751414

[12] Ahmad Shiekh Tanveer, Arjumand W, Nafees S, Seth A, Ali N, Rashid S. (2012) Hesperidinalleviates acetaminophen induced toxicity in wistar rats by abrogation of oxidative stress, apoptosis and inflammation.Toxicol Lett 208:149–161. 10.1016/j.toxlet.2011.10.023 22093918

[13] Salvioli S, Ardizzoni A, Franceschi C, Cossarizza A. JC-1, but not DiOC6(3) or rhodamine 123, is a reliable fluorescent probe to assess delta psi changes in intact cells: implications for studies on mitochondrial functionality during apoptosis. FEBS Lett 1997; 411:77–82. 9247146 10.1016/s0014-5793(97)00669-8

[14] Brooks MM, Neelam S, Fudala R, Gryczynski I, Cammarata PR.Lenticular mitoprotection. Part A: Monitoring mitochondrial depolarization with JC-1 and artifactual florescence by the glycogen synthase kinase-3β inhibitor, SB216763. Mol Vis. 2013 6 27;19:1406–12. 23825920 PMC3695757

[15] Perelman A, Wachtel C, Cohen M, Haupt S, Shapiro H, Tzur A.JC-1: alternative excitation wavelengths facilitate mitochondrial membrane potential cytometry. Cell Death Dis. 2012 11 22;3:e430 10.1038/cddis.2012.171 23171850 PMC3542606

[16] Park Y, Hirose R, Dang K, Xu F, Behrends M, Tan V, et al (2008) Increased severity of renal ischemia-reperfusion injury with venous clamping compared to arterial clamping in a rat model. Surgery 143(2):243–51. 10.1016/j.surg.2007.07.041 18242341

[17] Rehman J, Monga M, Landman J, Lee DI, Felfela T, Conradie MC, et al (2003) Characterization of intrapelvic pressure during ureteropyeloscopy with ureteral access sheaths. Urology 61(4): 713–8. 12670551 10.1016/s0090-4295(02)02440-8

[18] Low RK. (1999) Nephroscopy sheath characteristics and intrarenal pelvic pressure: human kidney model. J Endouro 13(3):205–8. 10.1089/end.1999.13.205 10360501

[19] de Geus HR, Betjes MG, Schaick Rv, Groeneveld JA. (2013) Plasma NGAL similarly predicts acute kidney injury in sepsis and nonsepsis. Biomark Med 7(3): 415–21. 10.2217/bmm.13.5 23734805

[20] Hervouet E, Simonnet H, Godinot C. (2007) Mitochondria and reactive oxygen species in renal cancer. Biochimie 89(9): 1080–8. 17466430 10.1016/j.biochi.2007.03.010

[21] Khattab MM, Mostafa A, Al-Shabanah O. (2005) Effects of captopril on car diac and renal damage, and metabolic alterations in the nitric oxide-deficient hypertensive rat. Kidney Blood Press Res 28: 243–250. 16220007 10.1159/000088829

[22] Yilmaz HR, Uz E, Yucel N, Ozcelik N. (2004) Protective effect of caffeic acid phenethyl ester (CAPE) on lipid peroxidation and antioxidant enzymes in diabetic rat liver. J Biochem Mol Toxicol 18(4): 234–8. 15452882 10.1002/jbt.20028

[23] Mortensen J, Shames B, Johnson CP, Nilakantan V. (2011) MnTMPyP, a superoxide dismutase/catalase mimetic, decreases inflammatory indices in ischemic acute kidney injury. Inflamm Res 60(3): 299–307. 10.1007/s00011-010-0268-3 21153678

[24] Kawakami T, Puri N, Sodhi K, Bellner L, Takahashi T, Morita K, et al Reciprocal Effects of Oxidative Stress on Heme Oxygenase Expression and Activity Contributes to Reno-Vascular Abnormalitiesin EC-SOD Knockout Mice. Int J Hypertens. 2012;2012:740203 10.1155/2012/740203 22292113 PMC3265091

[25] Reddi AR, Jensen LT, Naranuntarat A, Rosenfeld L, Leung E, Shah R, et al The Overlapping Roles of Manganese and Cu/Zn SOD in Oxidative Stress Protection. Free Radic Biol Med. 2009 1 15;46(2):154–62. 10.1016/j.freeradbiomed.2008.09.032 18973803 PMC2707084

[26] Jassem W, Fuggle SV, Rela M, Koo DD, Heaton ND. The role of mitochondria in ischemia/reperfusion injury. Transplantation. 2002 2 27;73(4):493–9. 11889418 10.1097/00007890-200202270-00001

[27] Zhu X, Smith MA, Perry G. Aliev G. Mitochondrial failures in Alzheimer's disease. American Journal of Alzheimer's Disease and Other Dementias 2004 Nov-Dec;19(6) 345–352. 15633943 10.1177/153331750401900611 PMC10834012

[28] Long J, Wang X, Gao H, Liu Z, Liu C, Miao M, et al Malonaldehyde acts as a mitochondrial toxin: Inhibitory effects on respiratory function and enzyme activities in isolated rat liver mitochondria. Life Sci. 2006 9 5;79(15):1466–72. 16737718 10.1016/j.lfs.2006.04.024

[29] Serel TA, Ozguner F, Soyupek S. (2004) Prevention of shock wave-induced renal oxidative stress by melatonin: an experimental study. Urol Res 32(1):69–71 14727104 10.1007/s00240-003-0397-z

[30] Cohen HJ, Tape EH, Novak J, Chovaniec ME, Liegey P, Whitin JC. The role of glutathione reductase in maintaining human granulocyte function and sensitivity to exogenous H2O2. Blood.1987; 69:493–500. 3801665

[31] Mehaney DA, Darwish HA, Hegazy RA, Nooh MM, Tawdy AM, Gawdat HI. et al Analysis of oxidative stress status, catalase and catechol-O-methyltransferase polymorphisms in Egyptian vitiligo patients. PLoS One. 2014 6 10; 9(6):e99286 10.1371/journal.pone.0099286 24915010 PMC4051781

[32] Kroemer G, Galluzzi L, Brenner C. (2007) Mitochondrial membrane permeabilization in cell death. Physiol Rev 87(1):99–163. 17237344 10.1152/physrev.00013.2006

[33] Davidson SM, Duchen MR. (2007) Endothelial mitochondria: contributing to vascular function and disease. Circ Res 100(8):1128–41. 17463328 10.1161/01.RES.0000261970.18328.1d

[34] Blanco FJ, López-Armada MJ, Maneiro E. (2004) Mitochondrial dysfunction in osteoarthritis. Mitochondrion 4(5–6):715–28. 16120427 10.1016/j.mito.2004.07.022

[35] Maneiro E, López-Armada MJ, de Andres MC, Caramés B, Martín MA, Bonilla A, et al (2005) Effect of nitric oxide on mitochondrial respiratory activity of human aricular chondrocytes. Ann Rheum Dis 64(3): 388–95. 15708893 10.1136/ard.2004.022152 PMC1755391

[36] Golstein P, Kroemer G. (2007) Cell death by necrosis: towards a molecular definition. Trends Biochem Sci 32(1):37–43. 17141506 10.1016/j.tibs.2006.11.001

[37] Halestrap, Andrew P. (2009) What is the mitochondrial permeability transition pore? J Mol Cell Cardiol 46(6):821–31. 10.1016/j.yjmcc.2009.02.021 19265700

[38] Dissing TH, Eskild-Jensen A, Pagh C, Frokiaer J, Rehling M, Jørgensen HS. et al (2001) Partial unilateral ureteropelvic junction obstruction induce in 2-week-old piglets. J Urol 166(6):2354–8. 11696784

[39] Wen JG. (2002) Partial unilateral ureteral obstruction in rats. Neurourol Urodyn 21(3):231–50. 11948717 10.1002/nau.10006

[40] Wåhlin N, Stenberg A, Persson AE. (2001) Renal blood flow increase during volume expansion in hydronephrotic rats. J Urol 165(5):1696–9. 11342958

